# Identifying biomarkers of dementia prevalent among amnestic mild cognitively impaired ethnic female patients

**DOI:** 10.1186/s13195-016-0211-0

**Published:** 2016-10-18

**Authors:** Rinko Grewal, Mona Haghighi, Shuai Huang, Amanda G. Smith, Chuanhai Cao, Xiaoyang Lin, Daniel C. Lee, Nancy Teten, Angela M. Hill, Maj-Linda B. Selenica

**Affiliations:** 1 Byrd Alzheimer’s Institute, University of South Florida, 4001 E. Fletcher Ave, Tampa, FL 33613 USA; 2 Department of Pharmaceutical Sciences, College of Pharmacy, University of South Florida, 12901 Bruce B. Downs Blvd, Tampa, FL 33612 USA; 3 Department of Pharmacotherapeutics and Clinical Research, College of Pharmacy, University of South Florida, 12901 Bruce B. Downs Blvd, Tampa, FL 33612 USA; 4 Department of Psychiatry and Behavioral Medicine, College of Medicine, University of South Florida, 3515 E Fletcher Ave, Tampa, FL 33613 USA; 5 Department of Industrial and Systems Engineering, University of Washington, 3900 Northeast Stevens Way, Seattle, WA 98195 USA; 6 School of Aging Studies, University of South Florida, 4202 E Fowler Ave, Tampa, FL 33620 USA

**Keywords:** Plasma, Biomarkers, Mild cognitive impairment, Amyloid beta, Cystatin C, Eotaxin-1, Female, Ethnicity

## Abstract

**Background:**

There is a need to investigate biomarkers that are indicative of the progression of dementia in ethnic patient populations. The disparity of information in these populations has been the focus of many clinical and academic centers, including ours, to contribute to a higher success rate in clinical trials. In this study, we have investigated plasma biomarkers in amnestic mild cognitively impaired (aMCI) female patient cohorts in the context of ethnicity and cognitive status.

**Method:**

A panel of 12 biomarkers involved in the progression of brain pathology, inflammation, and cardiovascular disorders were investigated in female cohorts of African American, Hispanic, and White aMCI patients. Both biochemical and algorithmic analyses were applied to correlate biomarker levels measured during the early stages of the disease for each ethnicity.

**Results:**

We report elevated plasma Aβ_40_, Aβ_42_, YKL-40, and cystatin C levels in the Hispanic cohort at early aMCI status. In addition, elevated plasma Aβ_40_ levels were associated with the aMCI status in both White and African American patient cohorts by the decision tree algorithm. Eotaxin-1 levels, as determined by the decision tree algorithm and biochemically measured total tau levels, were associated with the aMCI status in the African American cohort.

**Conclusions:**

Overall, our data displayed novel differences in the plasma biomarkers of the aMCI female cohorts where the plasma levels of several biomarkers distinguished between each ethnicity at an early aMCI stage. Identification of these plasma biomarkers encourages new areas of investigation among aMCI ethnic populations, including larger patient cohorts and longitudinal study designs.

**Electronic supplementary material:**

The online version of this article (doi:10.1186/s13195-016-0211-0) contains supplementary material, which is available to authorized users.

## Background

The use of biomarker data to detect early Alzheimer’s disease (AD) has significant benefits in therapeutic and diagnostic implications [1]. The new criteria and guidelines for diagnosis of AD and its early clinical stage of “Mild Cognitive Impairment due to AD” (MCI-AD) [2] and “Prodromal or amnestic AD” (aMCI) [3] highlights the importance of identifying early detection of specific and sensitive biomarkers as an effective disease intervention strategy [4, 5]. Cerebrospinal fluid (CSF) has been used to identify specific biomarkers that aid in the early detection of AD [6]. However, due to the invasive nature of CSF collection, the utilization of biological samples, such as plasma, presents a more feasible option [1, 7]. Studies have shown that high Aβ_42_ levels in plasma were linked to the low incidence of AD, while significant reduction of total tau levels in plasma was detected in AD patients, suggesting the viability of both proteins as biomarkers in AD [8, 9].

The study of supplemental cardiovascular and inflammatory biomarkers may also allow for an effective early diagnosis. This is especially relevant in ethnic populations, such as African American and Hispanic communities, where the prevalence for vascular diseases associated with high blood pressure and diabetes are higher with no known genetic factors explaining the increased incidence [10, 11]. Despite the association of cardiovascular risk factors with the increased risk of AD in African Americans, few studies have focused on the neuropathological association in this population [12–14]. Laboratory research and genome-wide association studies (GWAS) have recognized the impact of inflammation on AD pathology [15–17]. Indeed, a multitude of studies have investigated inflammatory markers as potential biomarkers in AD progression [18–20]; however, the data obtained from studies measuring levels of cytokines, cytokine receptors, and other proteins associated with immune responses in blood and CSF of AD patients are inconsistent. As our understanding on the impact of sex or ethnicity in disease progression increases, so should our efforts to design longitudinal and cross-sectional studies that extricate the progression of AD in well-controlled patient cohorts based on these parameters.

In this study, we aimed to identify biomarkers that link the incidence of aMCI/prodromal AD disease status to specific vascular, inflammatory, and other plasma biomarkers in ethnic female patient populations. We evaluated plasma samples of 75 female subjects from African American, Hispanic, and White ethnic backgrounds who were participants in the Florida Alzheimer’s Disease Research Center Clinical Core (FADRC-CC) in Miami Beach and Tampa, Florida [21]. All patients were classified as amnestic MCI (aMCI)/prodromal AD based on an approved consensus diagnosis as described previously [21, 22]. Further, we utilized computational analysis (i.e., a decision tree model) and correlational models to predict early AD plasma biomarkers present in each of the ethnic groups. We report elevated plasma levels of Aβ_40_, Aβ_42_, YKL-40, and cystatin C in the Hispanic AD cohorts by both biochemical and computational analyses. Increases in plasma Aβ_40_ levels were also demonstrated in aMCI patients in the White cohort, and a significant reduction in total tau levels were detected in the aMCI African American patient cohort. Further, with the algorithmic decision tree model we identified Aβ_40_ and eotaxin-1 as potential early indicators of AD progression in the African American female cohort. All together, these findings provide the necessary data for designing longitudinal studies where large ethnic groups can be included, and the biomarker selection can differentiate among ethnic groups and disease status.

## Methods

### Subjects and aMCI diagnosis

Plasma samples were obtained from the University of South Florida (USF) FADRC-CC database belonging to African American, Hispanic, and White female patients who were treated and evaluated at the Byrd Alzheimer’s Institute between the years 2006 and 2011. The FADRC-CC subjects had previously undergone neuropsychological tests as outlined in the National Alzheimer’s Coordinating Center (NACC) Uniform Data Set (UDS). Consesus diagnosis of the subjects was based on the physician's cognitive diagnosis, neuropsychological diagnosis, traditional consensus diagnosis, algorithmic diagnosis, and digital MRI scans [21]. Further, based on the FADRC-CC archival data, the Mini-Mental State Examination (MMSE), the Global Clinical Dementia Rating Scale (CDR), and neurophysiological assessment were also used for diagnosis [21]. For the classification of aMCI/prodromal AD, previously approved consensus was followed [21–23]. In addition, the *International Statistical Classification of Diseases, 10th Revision* and the *Diagnostic and Statistical Manual of Mental Disorders, Fifth Edition* criteria were met [24].

Of the 75 female ethnic subjects, 30 samples belonged to age-matched female controls (*n* = 10/group) and 45 samples belonged to female patients diagnosed with aMCI/prodromal AD disease stage (*n* = 15/group) based on the aforementioned diagnostic criteria [21, 22]. The White, African American, and Hispanic patient cohorts averaged an age of 76.23 ± 1.47 years (age range: 60–85 years) and maintained an averaged MMSE score of 26.88 ± 1.06 and CDR of 1.00 ± 0.5. The age-matched control population had an average age of 72.97 ± 1.60 and maintained an average MMSE value of 28.82 ± 0.45 and a CDR value of 0. The specific demographics of each ethnic cohort are presented in Table 1. Further, the level of education of each patient included in the study, which can be associated with patient cognitive reserve [25], and the patients’ ApoE genotype [26, 27] did not account for the cognitive status and other outcomes measured here. In addition, patients with disclosed cardiovascular and inflammatory conditions were used as exclusion criteria for this study. Plasma from all control and aMCI subjects was prepared according to the Clinical Laboratory Improvement Amendments (CLIA) standards, which deemed these samples suitable for clinical trial work.

**Table 1:** Demographics of female patients among the ethnic groups

	NC		aMCI		MMSE		CDR	
	Age (years) (± SEM)	*n*	Age (years) (± SEM)	*n*	NC	aMCI	NC	aMCI
White	73.40 ± 1.40	10	73.47 ± 1.17	15	28.71 ± 0.46	26.83 ± 1.58	0.00 ± 0.00	1.00 ± 0.39
African American	70.00 ± 1.86	10	76.73 ± 1.82	15	29.25 ± 0.31	27.15 ± 0.68	0.00 ± 0.00	1.00 ± 0.58
Hispanic	75.5 ± 2.04	10	78.50 ± 1.69	15	28.50 ± 0.56	26.67 ± 0.91	0.00 ± 0.00	1.00 ± 0.50

Plasma samples from African American, Hispanic, and White female patients diagnosed with aMCI (*n* = 15/ethnicity) and age-matched individuals (*n* = 10/ethnicity). Patients’ age (year), MMSE scores, and CDR values for each ethnic group are indicated as average (± SEM)

*aMCI* amnestic mild cognitive impairment, *MMSE* Mini-Mental State Examination *CDR* Global Clinical Dementia Rating Scale, *NC* age-matched control group

### Multiplex assay

The plasma samples were prepared with ethylenediaminetetraacetic acid (EDTA) based on the CLIA standards. Milliplex Multiplex assays, commercially available customized plates, were used with few modifications to the manufacturer’s instructions. The following plasma biomarkers were purchased from EMD Millipore: total tau (catalogue number HND1MAG-39 K), phosphorylated tau (catalogue number HND1MAG-39 K), C-reactive protein (catalogue number HCVD3MAG-67 K), fibrinogen (catalogue number HCVD3MAG-67 K), plasminogen activator/inhibitor (catalogue number HADK1MAG-61 K), MCP-1 (catalogue number HCYTOMAG-60 K), eotaxin-1 (catalogue number HCYTOMAG-60 K), and YKL-40 (catalogue number HCMBMAG-22 K). The standards, controls, and subject samples were incubated with fluorescent-coded magnetic MagPlex-C microspheres overnight at 4 °C, and additionally incubated with a biotinylated detection antibody for 1 hour at room temperature (RT). The streptavidin–phycoerythrin conjugate was added and incubated at RT for 30 minutes, and then rinsed three times. The plates were read using Luminex xMAP technology (Bio-Rad Laboratories, Inc., Hercules, CA, USA) where microspheres were counted based on fluorescent reporter signals.

### ELISA assay

R&D Systems Quantikine ELISA kits (R&D Systems, Inc., Minneapolis, MN, USA) measured progranulin (catalogue number DPGRN0) and cystatin C (catalogue number DSCTC0). The assay diluent was added to each well, precoated with antibody, prior to addition of standard, control, and subject samples, incubated for 2 hours at RT, and washed four times with wash buffer. An enzyme-linked human progranulin or human cystatin C conjugate was introduced into the wells. The plates were rinsed and incubated for 30 minutes at RT with substrate solution followed by the addition of a stop solution. The ELISA plates were read with the BioTek plate reader at wavelengths 540 nm and 570 nm and analyzed via the Gen5 program.

For determination of human plasma Aβ_40_ and Aβ_42_ levels, we used ELISA kits according to the manufacturer’s protocol (KHB3482 for Aβ_40_, KHB3442 for Aβ_42_; Invitrogen, Waltham, CA, USA). Optical density and concentrations were calculated according to the standard curve.

### Decision tree analysis

The decision tree model was developed as described recently [28]. The biomarker data gathered for each ethnic group are used to identify homogeneous subgroups embedded in each ethnicity whose members have similar biomarker levels. Model 1 (M1) describes a decision tree on data from African American female patients; Model 2 (M2) describes a decision tree on data from Hispanic female patients; and Model 3 (M3) describes a decision tree on data from White female patients. The decision trees are generated using conditional recursive partitioning, which recursively seeks cutoff values of the best biomarkers that can segment the population into homogeneous subgroups while the homogeneity of the subgroups is measured by the variation of the outcome variable. The final product of this method is a set of subgroups where each subgroup is defined as a rule that consists of multiple biomarkers and their cutoff values. In this method, we try to find rules that identify homogeneous subgroups of aMCI subjects or age-matched control individuals from the initial population. Specifically, as a recursive procedure, the algorithm grows the decision tree from the “root node” that includes all of the subjects. The algorithm then considers all of the possible cutoff values for each biomarker and uses univariate tests to find the best biomarker and its cutoff value to split the subjects in the root node into two partitions, resulting in two nodes. This split procedure is then repeated on each of the two nodes, further purifying the subgroups by splitting each node into two new nodes. This process is repeated until each node includes the predetermined number of individuals. When this process is terminated, the final subgroups or nodes would be the terminal nodes of the tree. We used the “party” package from R version 3.2.0 (http://www.r-project.org/) to develop the decision trees as a tool for classifying aMCI subjects and age-matched controls.

### Statistical analysis

All raw values of analytes measured with Milliplex Multiplex assays were adjusted using Milliplex Analyst 5.1 (Millipore, Billerica, MA, USA). In order to achieve normal distribution, the raw values of the analytes were adjusted by a standard curve (five-parameter logistic regression or five-parameter linear regression) that best fit the data. All raw values of analytes measured with R&D Systems Quantikine ELISA kits were adjusted using Elisa Analysis (2012). Normal distribution was achieved when the raw values were adjusted by the standard curve (five-parameter logistic regression) that best fit the data. Human plasma Aβ_40_ and Aβ_42_ levels using ELISA kits were adjusted using a best-fit standard curve created in Microsoft Excel (2010).

Statistical analysis in this study was performed in IBM SPSS 22.0 (IBM, Chicago, IL, USA). Using SPSS 22.0, a two by three factorial design with post-hoc analyses was used to compare age-matched controls and aMCI patients for each ethnicity. The pairwise comparison yield was used to determine significant changes between the normal controls and aMCI patients for each ethnic group (*p* ≤ 0.05). In IBM SPSS 22.0, an outlier test was performed, and data points that did not pass the test were excluded from further statistical analyses. Student’s *t*-test analysis was used to compare age-matched controls with aMCI patients (*p* ≤ 0.05). Graphs were generated using GraphPad Prism 5.0 (GraphPad Software, La Jolla, CA, USA).

## Results

### Ethnic patient and control population demographics and cognitive functioning criteria

There is a pressing need to find blood-based biomarkers that, with high sensitivity and specificity, can determine early AD risk and develop preventive therapies [29]. In our study, the aMCI patient population (*n* = 15/group) and age-matched controls (*n* = 10/group) consisted of White, African American, and Hispanic patients. The specific population demographics of each cohort and ethnicity are presented in Table 1.

Evidence suggests that levels of plasma Aβ_40,_ Aβ_42,_ total tau, phosphorylated tau, and cystatin C, in association with the development of neuropathology in AD brain, are well-established and internationally validated plasma biomarkers in AD [30–33]. Because their temporal detection levels are debated, we have included these potential biomarkers in this study [34–36]. In addition, vascular and inflammatory biomarkers are also potential factors that could provide a significant role in the early detection of AD [12, 37]. A list of all biomarkers measured in our cohort is presented in Table 2.

**Table 2:** Plasma biomarkers measured for each category reflecting neuropathological, vascular, and inflammatory implications

Biomarker	Type	Regulation in AD	References
Aβ_40_	Neuropathological	↑	Mehta et al., 2000 [45]
Aβ_42_	Neuropathological	↓	Craig-Schapiro et al., 2009 [56]
Total tau	Neuropathological	↑	Sunderland et al., 2003 [64]
Phospho-tau	Neuropathological	↑	Hampel and Teipel, 2004 [65]
Cystatin C	Neuropathological	↓	Sundelof et al., 2008 [55] Hu et al., 2012 [66]
C-reactive protein (CRP)	Vascular	↑	Yasojima et al., 2000 [67] Hu et al., 2012 [66] Cortes-Canteli et al., 2012 [68]
Fibrinogen	Vascular	↑	Hu et al., 2012 [66]
Plasminogen activator/inhibitor	Vascular	↑	Oh et al., 2014 [60]
MCP-10	Inflammatory	↑	Galimberti et al., 2006 [69]
Eotaxin 1G	Inflammatory	↑	Choi et al., 2008 [37]
YKL-40G	Inflammatory	↑	Craig-Schapiro et al., 2010 [39]
Progranulin G	Inflammatory	↑	D’Alton and Lewis, 2014 [70]

Biomarkers were chosen based on preclinical and clinical relevance to AD. *Arrows* indicate changes in biomarkers suggested from referred literature

*AD* Alzheimer’s disease

### Elevated plasma amyloid, tau, YKL-40, and cystatin C levels among female patients

**Fig. 1:**
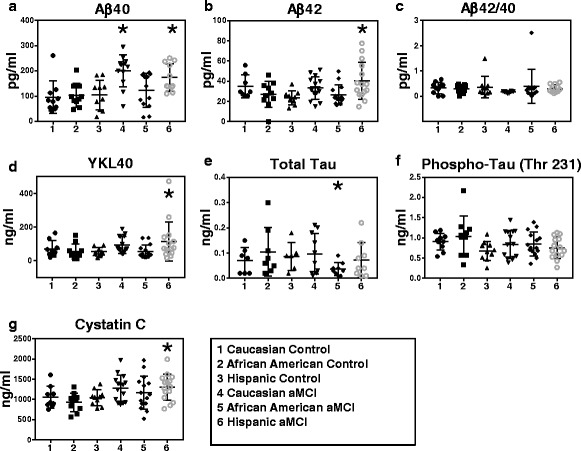
Plasma biomarkers revealing significant association with the incidence of aMCI in ethnic cohorts. **a** Aβ_40_ levels were significantly increased in White (*p* = 0.0001, 95 % CI: 58.0–134.0) and Hispanic (*p* < 0.01, 95 % CI: 66.8–142.8) aMCI patients compared with their respective age-matched control group (NC). **b** Plasma Aβ_42_ levels were significantly increased in the Hispanic aMCI patient population (*p* < 0.005, 95 % CI: 15.5–31.8) compared with the NC group. **c** Aβ_42_/Aβ_40_ ratio displayed no significant changes among aMCI ethnic groups. **d** YKL-40 levels were significantly increased in the aMCI group of the Hispanic population compared with its NC group (*p* < 0.05, 95 % CI: 10.7–97.7). **e** Plasma total tau levels were significantly associated with aMCI incidence in the African American aMCI patient cohort (*p* < 0.05, 95 % CI: 0.06–0.15), while **f** plasma phospho-tau levels remained unchanged independent of ethnicity and disease progression. **g** Plasma cystatin C levels in the Hispanic patient cohorts are associated with the incidence of aMCI (*p* < 0.05, 95 % CI: 844.8–1240.7). Statistical analyses were performed in a two by three factorial design with post-hoc analyses using IBM SPSS Statistics 22. An outlier test via IBM SPSS Statistics 22 was performed on each data set and outliers were removed. *aMCI* amnestic mild cognitive impairment

The analysis of biomarkers was conducted using plasma collected during patient visits. First, we measured levels of Aβ_40,_ Aβ_42_, and their ratio (Aβ_42_/Aβ_40_) in the plasma of each ethnic group and their respective normal age-matched controls (Fig. 1 and Additional file 1: Figure S1). The plasma Aβ_40_ levels displayed significant increases in the aMCI patient populations compared with the age-matched control group, independent of ethnicity (Fig. 1 and Table 3, *p* = 0.0001, Student’s *t* test). When controlled for ethnicity and disease status, our data analyses (IBM SPSS Statistics Desktop, V22.0) revealed pairwise significance (Fig. 1). Plasma Aβ_40_ levels were significantly increased in the aMCI female White patients (Fig. 1a and Table 4, *p* = 0.0001, 96.0 vs 160.5 pg/ml, 95 % CI: 58.0–134.0) and Hispanic patients (Fig. 1a and Table 4, *p* < 0.05, 104.8 vs 127.6 pg/ml, 95 % CI: 66.8–142.8) compared with their respective control groups. Further, increased plasma Aβ_42_ levels were significantly associated with the incidence of aMCI in the Hispanic patient population compared with its control (Fig. 1b and Table 4, *p* < 0.005, 23.7 pg/ml vs 40.4 pg/ml, 95 % CI: 15.5–31.8); however, this did not occur in either the White or African American patient cohorts. Plasma total tau levels were significantly decreased in the African American patient cohort (Fig. 1e and Table 4, *p* < 0.05, 0.11 vs 0.04 ng/ml, 95 % CI: 0.055–0.151) while its levels were unchanged in the Hispanic and White female patient cohorts. The levels of phosphorylated tau in plasma from all three patient cohorts remained unchanged at this early stage of dementia (Fig. 1f, Table 4, and Additional file 2: Table S1, SPSS statistical analysis).

**Table 3:** Statistical analysis of association between normal control and aMCI cohorts, independent of ethnicity

Biomarker	*p* value, NC vs aMCI
Aβ_40_	0.0001***
Cystatin C	0.0024***

Indicated plasma levels of Aβ_40_ and cystatin C levels were significantly increased in aMCI patients compared with the age-matched NCs, independent of ethnicity (***p = 0.0001 and ***p = 0.0024, respectively). Other biomarkers displayed no significant changes in the plasma levels between the NC group and aMCI female patients (data not shown, *p* > 0.05). Statistical analyses were performed using Student’s *t* test

*aMCI* amnestic mild cognitive impairment, *NC* age-matched control group

**Table 4:** Statistical analysis of association between normal control and aMCI ethnic cohorts

Biomarker	Ethnicity	*p* value, NC vs aMCI
Aβ_40_	White	0.0001***
	African American	0.4480
	Hispanic	0.0110*
Aβ_42_	White	0.7650
	African American	0.5100
	Hispanic	0.0020**
Aβ_42_/Aβ_40_	White	0.0800*
	African American	0.6570
	Hispanic	0.5240
Total tau	White	0.3700
	African American	0.0460*
	Hispanic	0.7070
Cystatin C	White	0.0970
	African American	0.0630
	Hispanic	0.0460*
YKL40	White	0.4180
	African American	0.9880
	Hispanic	0.0330*

Indicated are the levels of biochemically measured plasma biomarkers that displayed significant changes controlled for ethnicity and compared with NC cohort. Statistical analyses were performed in a two by three factorial design with post-hoc analyses using IBM SPSS Statistics 22. An outlier test was also performed using IBM SPSS Statistics 22. Significant changes in biomarkers related to ethnic background: *p < 0.05, **p < 0.01 ***p < 0.001

*aMCI* amnestic mild cognitive impairment, *NC* age-matched control group

Studies have associated cystatin C levels with the pathology of AD [31]. Our data demonstrated significant increases in cystatin C levels in the plasma of aMCI patients from all three patient cohorts compared with age-matched controls (Fig. 1g and Table 3, Student’s *t* test, *p* < 0.005). When corrected for ethnicity and disease status, plasma levels of cystatin C were significantly higher in Hispanic aMCI female patients compared with the Hispanic age-matched control group (Fig. 1g, *p* < 0.05, 1042.7 vs 1303.4 ng/ml, 95 % CI: 844.8–1240.7). All collected data are summarized in Additional file 2: Table S1.

### Plasma inflammation and vascular biomarker associations in female patients

Inflammatory biomarkers are of interest due to the association of proinflammatory cytokines in the cascade leading to the progression of plaques and neurofibrillary tangle pathology in AD [38]. Utilizing the multiplex technology, we found a significant increase in YKL-40 levels in the plasma of the Hispanic aMCI patient cohort compared with its respective age-matched control (Fig. 1d, *p* < 0.05, 54.2 vs 114.1 ng/ml, 95 % CI: 10.7–97.7). Interestingly, studies have shown increased levels of YKL-40 in very mild and mild AD-type dementia compared with control subjects [39], suggesting the validity of YKL-40 as an early biomarker in general patient AD populations and specifically in those of Hispanic ethnicity.

The progression of AD and vascular diseases are highly increased with age [40]. Studies suggest that cerebrovascular disease and high cholesterol levels may be associated with AD [13, 14], and they relate to vascular conditions that are otherwise prominent risk factors for AD in African Americans [41, 42]. Therefore, plasma levels for vascular risk factors such as fibrinogen, plasminogen activator/inhibitor (PAI.1), and C-reactive protein (CRP) were measured in aMCI and aged-matched patient cohorts (Additional file 1: Figure S1). Statistical analysis revealed no significant difference in the plasma levels of vascular biomarkers between the control and patient cohorts controlled for ethnicity and cognitive status (Additional file 1: Figure S1 and Additional file 2: Table S1).

### Decision tree analysis identifies significant correlation between various markers and amyloid levels in the plasma of female patients

**Fig. 2:**
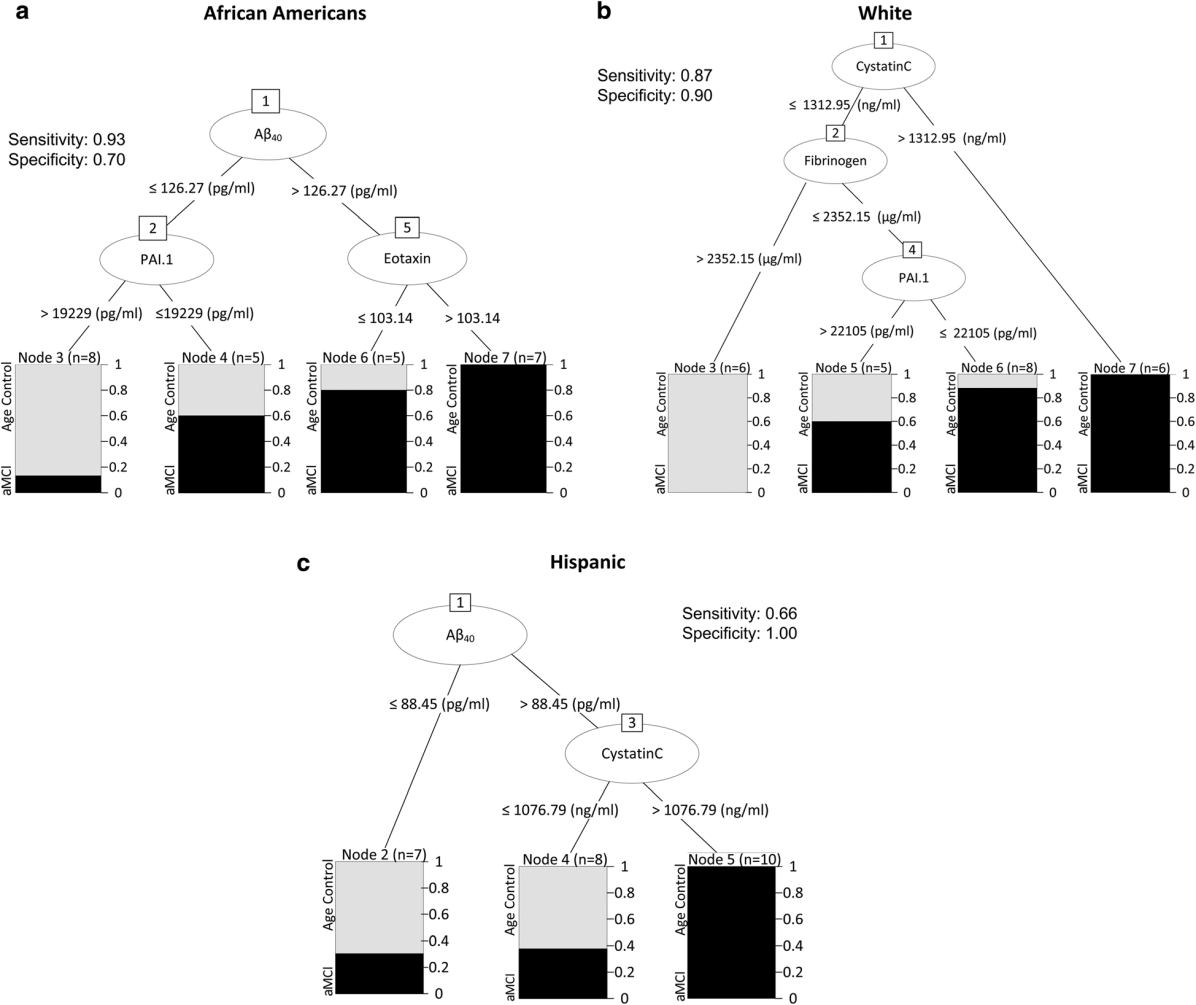
Decision tree models for each ethnic group represent biomarkers linked to the risk of developing AD. Each node represents the subgroups of patients obeying the rule of similar biomarker levels linked to the probability of developing aMCI for the given sample size. The sensitivity and specificity for each decision tree was determine

Next, we applied the decision tree (described in Methods), a computational modeling algorithm, to our data set in order to identify homogeneous subgroups with similar biomarker levels that link them to the risk of developing AD in each ethnic group (Fig. 2). The decision tree model applied for the African American female cohort (M1) identified three biomarkers—Aβ_40_, plasminogen activator/inhibitor (PAI.1), and eotaxin-1—which can characterize two homogeneous subgroups as Node 3 and Node 7. Node 3 is based on M1_Rule1 (Aβ_40_ ≤ 126.37 pg/ml and PAI.1 > 19229 pg/ml), which associates high levels of PAI.1 with increased age. Node 7 identifies a homogeneous subject group (*n* = 7), which can be characterized following M1_Rule2: Aβ_40_ > 126.27 pg/ml and eotaxin-1 > 103.14 pg/ml (Fig. 2a). Interestingly, Aβ_40_ and eotaxin-1 were most strongly associated with aMCI in the African American patients with plasma levels above 126.27 pg/ml and 103.14 pg/ml, respectively (Fig. 2a).

M2 model of the white female cohort identified three biomarkers; cystatin C, fibrinogen, and PAI.1, which were linked linked to 3 subgroups following the rule: M2_Rule1 identifies Node 3 as a homogeneous age-matched control group characterized as cystatin C ≤ 1312.95 ng/ml and fibrinogen > 2352.15 μg/ml; M2_Rule2 identifies Node 6 as a heterogeneous population of the aMCI patient group (*n* = 7/8 aMCI) characterized as cystatin C ≤ 1312.95 ng/ml, fibrinogen ≤ 2352.15 μg/ml, and PAI.1 ≤ 22105 pg/ml; and M2_Rule3 identifies Node 7 as a homogeneous aMCI subject group characterized as cystatin C > 1312.95 ng/ml (Fig. 2b).

M3 was built on the Hispanic cohort dataset and identified Aβ_40_ and cystatin C as biomarkers linked to one homogeneous subgroup of aMCI subjects (Fig. 2c). Node 5 is characterized by M3_Rule1: Aβ_40_ > 88.5 pg/ml and cystatin C > 1076.79 ng/ml. Interestingly, when the cystatin C rule in this decision tree was applied to the other two ethnicities, the majority of White and African American individuals who satisfy this rule were also classified as aMCI (data not shown). This suggests that cystatin C is associated with aMCI and should be studied further as a potential early detector of AD.

For each model, the sensitivity measured the ability of the model to identify the aMCI subjects, and the specificity determined the ability of the model to identify actual age-matched controls. Both sensitivity and specificity of M1 (93 % and 70 %, respectively) and M2 (87 % and 90 %, respectively) are satisfactory. M3 has a sensitivity of only 66.7 % and was unable to detect a homogeneous aMCI subgroup, although this model was performed with 100 % specificity. Overall, all three models displayed strong associations with aMCI despite the low sample size (*n* = 75) [43].

## Discussion

In this study, we analyzed a series of pathological, vascular, and inflammatory biomarkers in the plasma of African American, White, and Hispanic female aMCI patients and age-matched control individuals. The goal of this study was to determine effective potential biomarkers that are specific for each ethnicity and can be used for early detection of AD. Based on the multiplex biochemical findings, we applied a set of algorithmic rules which enabled us to identify biomarkers that are associated with AD progression. Because of the significant role of extracellular Aβ levels in the progression of AD [44], we investigated its plasma levels as a potential biomarker of aMCI disease stage in the study. An overall significant increase of Aβ_40_ plasma concentrations compared with the normal controls was shown. Plasma Aβ_40_ was also significantly increased in the White (*p* = 0.0001) and Hispanic (*p* < 0.05) aMCI patient cohorts, while plasma Aβ_42_ levels were significantly increased in the Hispanic aMCI patient cohort compared with its age-matched control (*p* < 0.005). Furthermore, the decision tree model identified that Aβ_40_ plasma levels were associated with the aMCI status of the African American female cohort. Studies have demonstrated conflicting results regarding amyloid levels in plasma, where both increases [45] or no significant changes [8] in Aβ_40_ levels between AD patients and normal controls have been reported. The discrepancies in the amyloid levels measured in these studies could reflect several issues that many laboratories face with sample treatment and analytical assays utilized for measurement of Aβ in plasma. A detailed discussion on preanalytical methods and protocols to ensure consistency among studies has been outlined in the new guidelines [46]. Further, contribution of several differential genetic factors, differences in prevalence of nongenetic medical risk factors, social reaction to cognitive decline, and environmental risk factors represent ultimately the statistical limitation of many reported studies.

Accumulation of hyperphosphorylated tau into neurofibrillary tangles in the brain is highly correlated with clinical manifestations and memory loss of AD patients [47]. This study found a significant decrease in the levels of total tau in the African American female aMCI stage patients compared with the age-control group, with no changes in phosphorylated tau levels among all ethnicities. Zetterberg et al. [30] found that plasma total tau levels were significantly elevated in AD patients, but not in MCI patients, when compared with normal controls. The authors suggested that plasma total tau levels may be elevated to abnormal levels in the later disease stages, and therefore plasma tau would be considered a late biomarker [30]. The lack of significant changes reported in the aforementioned studies could be due to overall low concentrations of total tau in plasma as well as the limitations that each analytical assay represents [48]. The disparity of data associated within each specific ethnicity and/or sex could also contribute to the discrepancies between published reports.

**Fig. 3:**
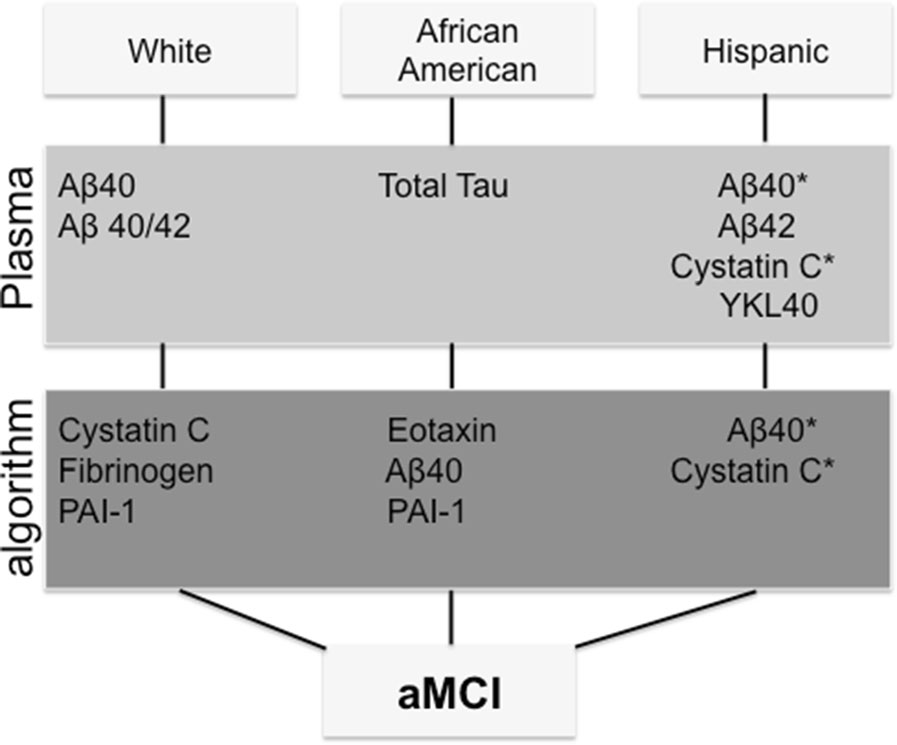
Biomarkers modulated in the plasma of female cohorts and associated with aMCI cognitive status and ethnicity. *Biomarkers that were similar across biochemical and algorithmic analysis. Notice the presence of cystatin C and Aβ_40_ across analysis in White and Hispanic patient cohorts (plasma vs algorithm). *aMCI* amnestic mild cognitive impairment, *PAI.1* plasminogen activator/inhibitor

Cystatin C is highly relevant to the progression of AD [49]. While a case–control genetic association study has linked the polymorphism of the cystatin C gene with the risk for late-onset AD [50], others have demonstrated that cystatin C polymorphism is a risk factor in early-onset AD [51]. In fact, early research links cystatin C with Aβ found in the vascular walls and senile plaque cores in the brains of patients with AD [52]. An amount of evidence suggests that cystatin C could even protect the brain against amyloid-induced toxicity by binding to Aβ protein and inhibiting Aβ_42_ oligomer and fibril formation [52–54]. A study examining an AD patient cohort consisting of older men aged 77 years found that the reduction of serum cystatin C levels was significantly associated with the increased risk of AD [55]. In our study, cystatin C levels were significantly increased in the aMCI female population compared with the age-matched controls. Further, we report that cystatin C was significantly higher in Hispanic aMCI female patients compared with the Hispanic age-matched control group (*p* < 0.05). The computational analysis on the Hispanic cohort dataset identified cystatin C as a biomarker linked to a homogeneous aMCI subgroup, suggesting that cystatin C can play an important role in the early detection of AD. Interestingly, when this algorithm (cystatin C > 1076.79 ng/ml) was applied to the White and African American cohorts, the majority of subjects that satisfied the rule were aMCI patients. Altogether, our finding adds to the growing body of literature suggesting that cystatin C may modulate the clinical progression and cognitive decline in the disease and can be suggested as a therapeutic biomarker for the early detection of AD (Fig. 3) [31].

We demonstrated that plasma levels of YKL-40 were elevated in the Hispanic aMCI female patients compared with the respective age-matched controls (*p* < 0.05). Interestingly, inflammatory markers including MCP-1 and progranulin were not significantly modified in the female patient cohort (independent of ethnicity), despite the potential of these markers as early detectors in the diagnosis of AD. The significant increase in YKL-40, a marker for neuroinflammation, in the Hispanic aMCI cohort is of great interest because YKL-40 elevation in both the CSF and plasma of MCI and mild AD-type dementia patients has been reported [56, 57]. However, a recent study suggested that increased YKL-40 levels in CSF does not provide higher diagnostic accuracy compared with CSF Aβ and tau levels [58], but notably reflecting the ongoing synaptic degeneration and glial activation in AD. Our findings corroborate the claim that YKL-40 can be a viable biomarker for early detection of AD, especially when controlled for ethnicity and sex.

Furthermore, the decision tree model identified Aβ_40_ and eotaxin-1 levels that were associated with aMCI in the African American female cohort. Interestingly, a human whole-genome study identified that a haplotype of SNPs on chromosome 17, a chemokine gene cluster (which includes eotaxin-1), was associated with age of onset in familial AD [59]. Another study measuring chemokine and cytokine levels in a cohort consisting of 13 controls and 11 AD patients reported significant elevation of serum eotaxin-1 levels in aMCI patients compared with the control group; however, the study did not account for differences between ethnic groups in their patient cohort [37]. Hereby, our findings suggest that increased eotaxin-1 plasma levels in the African American female population could reflect early changes in this cohort, a relationship that has yet to be studied.

Despite the nonsignificant changes measured in the cardiovascular biomarkers levels in our patient cohort, computational analysis identified that the combination of cystatin C ≤ 1312.95 ng/ml, fibrinogen ≤ 2352.15 μg/ml, and PAI.1 ≤ 22105 pg/ml is associated with 87 % of White female aMCI patients. One limitation in applying these analyses is the low sample size of patients enrolled in this study; therefore, the models applied here are only suggestive of relevant biomarkers. Cardiovascular markers have been shown to be modified during AD progression [13, 14]; for example, Oh et al. [60] reported increased levels of plasma PAI-1 levels in MCI and AD subjects as compared with normal controls. The authors also reported that the PAI-1 levels were gradually increased as the dementia progressed [60]. The discrepancy in our findings and those reported may be due to various methodological and analytic differences, including the patients’ ethnic background, sex, and medication history and the temporal expression profile of vascular factors. These factors and others can, for example, interfere with the biomarker plasma concentration [14].

Because GWAS confirmed the APOE e4 allele as a risk factor and identified ABC7, a membrane transporter protein, to be a strong genetic risk factor of AD in African Americans [61], both genes represent important correlative risk factors for future studies in the context of ethnicity and cognitive performance. We believe that finding biomarkers which link biological risk factors to cognitive function and determining biomarker disparity among ethnic groups can significantly advance research for the development of effective preventive therapeutic interventions in the treatment of AD.

## Conclusion

We report that elevated plasma Aβ_40_, Aβ_42_, cystatin C, and YKL-40 levels were associated with aMCI incidence in the Hispanic cohorts by both biochemical and computational analyses. Statistical analyses also showed that plasma Aβ_40_ and total tau levels were associated with the incidence of aMCI in the White cohort and African American cohort, respectively. By applying algorithmic rules in a decision tree model, we identified Aβ_40_ and eotaxin as potential early indicators of AD in the African American aMCI patient cohort, based on their sex and ethnicity. To our knowledge, very few studies have analyzed the relation of ethnicity to AD in such an inclusive panel of biomarkers. Considering that clinical manifestations are often the result of complex extrinsic social factors, there is growing awareness that these factors may influence some of the disparities in clinical presentation and treatment [62, 63].

## Additional files

Additional file 1: Figure S1. Plasma biomarkers that did not significantly change among ethnicities and disease status. Graphs represent six out of 12 biomarkers that were not significantly changed among ethnic groups with aMCI disease status compared with age-matched controls (NC). Statistical analyses were performed in a two by three factorial design with post-hoc analyses using IBM SPSS Statistics 22. An outlier test via IBM SPSS Statistics 22 was performed on each data set and outliers were removed. (TIFF 1521 kb)
Additional file 2: Table S1. Compiled values for all biomarkers measured in the plasma of aMCI vs NC subjects. Data for the measured levels of each biomarker (units indicated) within the ethnic group for aMCI (n = 15) and NC (n = 10) cohorts. Values are presented as average and standard error mean value (± SEM). (DOCX 104 kb)

## Abbreviations

MCI: Mild cognitive impairment
aMCI: Amnestic MCI
AD: Alzheimer’s disease
FADRC-CC: Florida Alzheimer’s Disease Research Center Clinical Core
NACC: National Alzheimer’s Coordinating Center
UDS: Uniform Data Set
GWAS: Genome-wide association studies
MMSE: Mini-Mental State Examination
CDR: Global Clinical Dementia Rating Scale
PAI.1: Plasminogen activator/inhibitor
CRP: C-reactive protein
Aβ: Amyloid beta
MCP-1: Monocyte chemoattractant protein-1
EDTA: Ethylenediaminetetraacetic acid
CLIA: Clinical Laboratory Improvement Amendments
