# Supplementary figures and images for: Identifying biomarkers of dementia prevalent among amnestic mild cognitively impaired ethnic female patients
Source: Alzheimers Res Ther. 2016 Oct 18;8:43. doi: 10.1186/s13195-016-0211-0 (PMC5067885; doi:10.1186/s13195-016-0211-0)

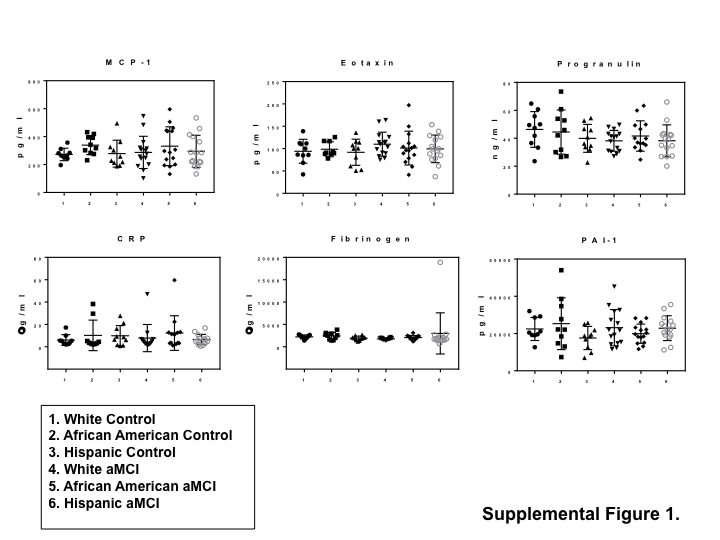

Supplement: Additional file 1: Figure S1. — Plasma biomarkers that did not significantly change among ethnicities and disease status. Graphs represent six out of 12 biomarkers that were not significantly changed among ethnic groups with aMCI disease status compared with age-matched controls (NC). Statistical analyses were performed in a two by three factorial design with post-hoc analyses using IBM SPSS Statistics 22. An outlier test via IBM SPSS Statistics 22 was performed on each data set and outliers were removed. (TIFF 1521 kb) [file 13195_2016_211_MOESM1_ESM.tiff]
