# Supplementary material for: Identifying biomarkers of dementia prevalent among amnestic mild cognitively impaired ethnic female patients
Source: Alzheimers Res Ther. 2016 Oct 18;8:43. doi: 10.1186/s13195-016-0211-0 (PMC5067885; doi:10.1186/s13195-016-0211-0)
Supplement: Additional file 2: Table S1. — Compiled values for all biomarkers measured in the plasma of aMCI vs NC subjects. Data for the measured levels of each biomarker (units indicated) within the ethnic group for aMCI (n = 15) and NC (n = 10) cohorts. Values are presented as average and standard error mean value (± SEM). (DOCX 104 kb) [file 13195_2016_211_MOESM2_ESM.docx]

**Additional file 2: Table S1** Biomarker levels measured in the plasma of aMCI vs. NC subjects
